# EHMTI-0216. Primary headache among adolescents in Albania

**DOI:** 10.1186/1129-2377-15-S1-B17

**Published:** 2014-09-18

**Authors:** J Kruja, I Alimehmeti, D Dobi, A Kuqo, S Mijo, S Grabova, M Rakacolli

**Affiliations:** 1 Neurology, University of Medicine, Tirana, Albania; 2 Statistic, Health Research and Development Center Tirana, Tirana, Albania; 3 Neurology, UHC Mother Teresa, Tirana, Albania

## Aim

To point out the prevalence of headache in adolescents.

## Methods

A door-to-door survey was undertaken in Tirana. The random samples of the local population underwent a structured interview to ascertain some neurological disorders, including headache. The diagnosis was made using standard criteria for epidemiological studies and was confirmed by history, neurological examination and where available, the review of personal medical records. The prevalence ratios according to age groups with 95% confidence intervals were calculated. The 15-19 years old group is studied in details. Age adjusted study to the general population age gropus is applied.

## Results

4.06% (n=51) of individuals reporting headache and living in urban areas are 15-19 years old. Comparing this figure to data extracted from the national census, where the proportion of 15-19 years old in the general urban population is 8.69% (n=130.227), generates a relative risk of 0.467 for having a headache in this age group. There is an extremely statistic significance in the difference between these two proportions (z=5.83, p<0.0001).

## Conclusion

Headache is relatively uncommon in 15-19 years old individuals in Tirana, Albania

No conflict of interest.

